# Cell cycle progression score as a predictive biomarker for overall survival in patients with adrenocortical carcinoma

**DOI:** 10.1002/ctm2.138

**Published:** 2020-07-23

**Authors:** Jing Sun, Run Shi, Felix Beuschlein, Belka Claus, Minglun Li

**Affiliations:** ^1^ Department of Radiation Oncology University Hospital Ludwig‐Maximilians‐Universität Munich Munich Germany; ^2^ Medizinische Klinik und Poliklinik IV Klinikum der Universität Ludwig‐Maximilians‐Universität München Munich Germany; ^3^ Klinik für Endokrinologie Diabetologie und Klinische Ernährung Unviersitätsspital Zürich Zurich Switzerland

Dear Editor,

Following the report by Cuzick et al on prognostic value of an established cell cycle progression score (CCPS) in prostate cancer,^1^ many studies have validated CCPS in further independent prostate cancer cohorts.^2^ Similarly, Cruzick's CCPS was found to predict mortality in other urological malignancies such as kidney cancer.^3^

The score is calculated upon the relative expression levels of 31 selected CCP genes normalized to 15 housekeeper genes using quantitative RT‐PCR. However, in this method some shortcomings are inevitable: First, these 31 genes, selected from a set of documented CCP genes using Pearson's correlation coefficient, have been disputed to comprehensively represent the hallmark of cell cycle progression. Second, the results of quantitative RT‐PCR are susceptible to considerable variability, including different RNA extraction methods, inconsistent human operations, and heterogeneous samples quality in repeated experiments. In the current study, we aimed to overcome these shortcomings, and have evaluated a novel method in patient cohorts with adrenocortical carcinoma (ACC).

In this study, single sample gene set enrichment analysis (ssGSEA)^4^ was performed to quantify CCP along with some other cancer‐related hallmarks such as “epithelial–mesenchymal transition (EMT),” “angiogenesis,” and “hypoxia,” based on corresponding gene sets retrieved from Molecular Signatures Database (MSigDB)^5^ and RSEM‐normalized RNA‐seq data of 79 ACC samples from The Cancer Genome Atlas (TCGA).^6^ Cibersort was used to quantify the immune infiltration based on the gene expression profile.^7^ *Z*‐score method was used to normalize both ssGSEA and immune infiltration scores. Distance between different hallmarks was depicted using hierarchical clustering analysis. Cox proportional‐hazards model was used to evaluate the importance of each hallmark for overall survival (OS). Clinical phenotype and somatic mutation data were used to depict relationships between ssGSEA‐derived CCPS and different clinicopathological features. One‐way analysis of variance (ANOVA) was performed to evaluate the difference of CCPS in different tumor stages. Kaplan‐Meier curve was plotted, and log‐rank test was used to evaluate survival difference between CCPS‐low and CCPS‐high group. Multivariate Cox regression analysis was performed to identify independent risk factors for OS among various clinicopathological variables including CCPS, age, gender, pathological T (pT) stage, pathological N (pN) stage, clinical M (cM) stage, and surgical margin (SM) status. Furthermore, the predictive performances of each parameter were compared at different time points in the follow‐up using time‐dependent receiver operating characteristic (tROC) with R package “survivalROC.”^8^

Distance between the nine cancer‐related hallmarks was shown in the cluster dendrogram in Figure 1A. We observed that CCP and “DNA repair” remain close to each other but distant to other hallmarks. Among various cancer‐related hallmarks, CCP exhibited the most powerful risk for OS (Figure 1B). The overview of relationships between CCPS and clinicopathological features demonstrated that CCPS was significantly correlated with factors considered to be associated with a more aggressive behavior such as heavy mutation burden, advanced tumor stages and worse clinical outcomes (Figure 1C, left panel). Considering cell cycle process being tightly correlated with tumor growth, we focused further investigation on the expression profile in different pT stages. As expected, CCPS was significantly and stepwisely elevated in more advanced pT stages (*P *= 1.01 × 10^‐5^; Figure 1C, right panel). Using the median as cutoff value, patients with higher CCPS exhibited significantly worse OS (HR = 12.91, 95% CI = 6.047‐27.57, *P *= 4.05 × 10^‐8^; Figure 1D). Multivariate Cox regression analysis demonstrated that only CCPS serves as an independent risk factor for OS among various clinicopathological features (HR = 4.721, 95% CI = 2.017‐11.05, *P *= 3.48e‐4; Figure 1E). In addition, tROC analysis indicated that CCPS can serve as a more powerful and consistent predictor for OS in a long‐term follow‐up, in comparison to other traditional prognostic parameters (Figure 1F).

**FIGURE 1:**
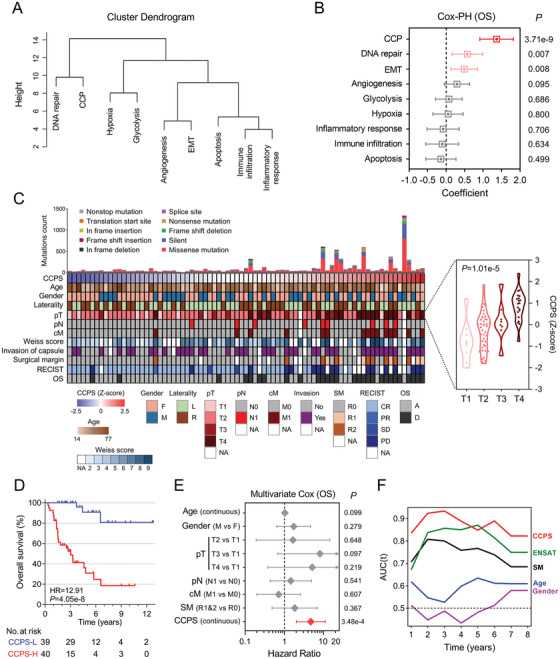
CCPS serves as a powerful risk factor and a promising predictor for OS in ACC. A, Cluster dendrogram depicts the distance between different hallmarks. B, Among various cancer‐related hallmarks, CCP is the strongest risk factor for overall survival in ACC. C, Overview of relationships between CCPS and clinicopathological features. Annotations: F, female; M, male; L, left; R, right; SM, surgical margin; RECIST, Response Evaluation Criteria in Solid Tumors; CR, complete response; PR, partial response; SD, stable disease; PD, progressive disease; A, alive; D, deceased; NA, not available. D, Patients with higher CCPS exhibited significantly worse prognosis. Annotations: CCPS‐L, CCPS‐low; CCPS‐H, CCPS‐high. E, Multivariate Cox regression analysis demonstrated that CCPS served as an independent risk factor for overall survival among various clinicopathological features. F, Results of tROC analysis demonstrated that CCPS served as a powerful and consistent predictor for overall survival in a long‐term follow‐up

In the current analysis, ssGSEA algorithm was used to quantify CCP. ssGSEA calculates separate enrichment scores for each pairing of a sample and gene set, thereby representing the degree to which the genes in a particular gene set are coordinately up‐ or down‐regulated within a sample.^4^ Based on these data, the ssGSEA‐derived CCPS serves as a powerful risk factor and a promising predictor for OS in ACC. Although this method seems robust and effective, validation in more studies, especially in prospectively designed trials, is warranted.

## CONFLICT OF INTEREST

The authors have declared no conflict of interest.
